# The endonuclease EEPD1 mediates synthetic lethality in RAD52-depleted BRCA1 mutant breast cancer cells

**DOI:** 10.1186/s13058-017-0912-8

**Published:** 2017-11-16

**Authors:** Robert Hromas, Hyun-Suk Kim, Gurjit Sidhu, Elizabeth Williamson, Aruna Jaiswal, Taylor A. Totterdale, Jocelyn Nole, Suk-Hee Lee, Jac A. Nickoloff, Kimi Y. Kong

**Affiliations:** 1 Department of Medicine and the Cancer Center, University of Florida Health, 1600 SW Archer Rd, Gainesville, FL 32610 USA; 2 Department of Biochemistry and Molecular Biology, Indiana University School of Medicine, Indianapolis, IN 46202, USA; 3 Department of Environmental and Radiological Health Sciences, Colorado State University, Fort Collins, CO 80523 USA

**Keywords:** Homologous recombination, Replication stress, Non-homologous end joining, synthetic lethality, BRCA1, Breast cancer

## Abstract

**Background:**

Proper repair and restart of stressed replication forks requires intact homologous recombination (HR). HR at stressed replication forks can be initiated by the 5′ endonuclease EEPD1, which cleaves the stalled replication fork. Inherited or acquired defects in HR, such as mutations in breast cancer susceptibility protein-1 (BRCA1) or BRCA2, predispose to cancer, including breast and ovarian cancers. In order for these HR-deficient tumor cells to proliferate, they become addicted to a bypass replication fork repair pathway mediated by radiation repair protein 52 (RAD52). Depleting RAD52 can cause synthetic lethality in BRCA1/2 mutant cancers by an unknown molecular mechanism.

**Methods:**

We hypothesized that cleavage of stressed replication forks by EEPD1 generates a fork repair intermediate that is toxic when HR-deficient cells cannot complete repair with the RAD52 bypass pathway. To test this hypothesis, we applied cell survival assays, immunofluorescence staining, DNA fiber and western blot analyses to look at the correlation between cell survival and genome integrity in control, EEPD1, RAD52 and EEPD1/RAD52 co-depletion BRCA1-deficient breast cancer cells.

**Results:**

Our data show that depletion of EEPD1 suppresses synthetic lethality, genome instability, mitotic catastrophe, and hypersensitivity to stress of replication of RAD52-depleted, BRCA1 mutant breast cancer cells. Without HR and the RAD52-dependent backup pathway, the BRCA1 mutant cancer cells depleted of EEPD1 skew to the alternative non-homologous end-joining DNA repair pathway for survival.

**Conclusion:**

This study indicates that the mechanism of synthetic lethality in RAD52-depleted BRCA1 mutant cancer cells depends on the endonuclease EEPD1. The data imply that EEPD1 cleavage of stressed replication forks may result in a toxic intermediate when replication fork repair cannot be completed.

**Electronic supplementary material:**

The online version of this article (doi:10.1186/s13058-017-0912-8) contains supplementary material, which is available to authorized users.

## Background

DNA replication does not proceed in a continuous manner, but stalls and restarts at sites of DNA damage [1–3]. DNA damage occurs continuously from both endogenous and exogenous sources [1–3]. Replication stress occurs when the rate of proliferation overtakes the clearance of the DNA damage ahead of progressing replication forks [1–3]. Cancer cells experience high levels of replication stress. Thus, efficient restart of stalled or collapsed replication forks is critical to their survival, particularly in response to common cancer therapeutics [4, 5]. Radiation repair protein 51 (RAD51)-dependent homologous recombination (HR) is the canonical repair and restart pathway for stalled replications forks [6–9]. HR is best characterized for the repair of DNA double-strand breaks (DSBs). HR is mediated by a litany of components that are regulated by breast cancer susceptibility protein-1 (BRCA1), which promotes the initial step in HR, 5′ end resection to create 3′ single-stranded (SS) DNA. BRCA2 then loads RAD51 onto this SS DNA to catalyze strand invasion into homologous sequences (typically the sister chromatid) creating heteroduplex DNA intermediates [2, 7, 8, 10]. After the invading strand re-initiates DNA replication, HR intermediates such as Holliday junctions are resolved by Holliday junction 5′ flap endonuclease (GEN1) or MUS81 structure-specific endonuclease subunit (MUS81), with SLX4 structure-specific endonuclease subunit (SLX4) serving as a scaffold [11–13].

HR repair of stressed replication forks also requires 5′ end resection as an initial step. This 5′ end resection needs a free DNA double strand (DS) end structure for the 5′ exonuclease activity in end resection. There are two ways to create this DS end: fork reversal to a chicken foot structure, or fork cleavage by a structure-specific nuclease [14–16]. We previously reported that the 5′ endonuclease EEPD1 could cleave stalled replication forks, initiate EXO1-mediated 5′ end resection, and promote repair of HR replication forks independent of BRCA1 [17–19]. However, BRCA1/2-mutant cancer cells lack HR, and how these cells repair stalled replication forks has been an unresolved issue. Several reports point to RAD52 in fulfilling this function. In yeast, Rad52 plays an essential role in HR, including HR-mediated restart of collapsed replication forks [20–22]. However, early studies suggested that in mammals the essential roles for RAD52 in HR have been supplanted [23], perhaps by BRCA2. We reported that human RAD52 foci appear 4–6 h after exposure to ionizing radiation, long after RAD51 foci appear, and we proposed that these late-appearing foci reflected a conserved role for human RAD52 in HR-mediated repair of collapsed replication forks [24]. This model was supported by a subsequent study showing that DSBs arising many hours after exposure to ionizing radiation were replication-dependent and repaired by HR [25]. In a separate line of investigation, RAD52 was identified as essential for viability of cancer cells with defects in various HR proteins including BRCA1, BRCA2, and partner and localizer of BRCA2 (PALB2) [23, 26, 27]. Together, these results suggest that RAD52 functions in a backup HR pathway independent of BRCA1/2, in which RAD52 loads RAD51 onto SS DNA for HR repair at stalled forks [26–29]. RAD51 then promotes the strand invasion required to complete HR repair and replication fork restart [26–28].

However, RAD52 still needs an end-resected 3′ SS upon which to load RAD51. Thus, 5′ end resection is still required for the backup pathway, yet this is problematic if BRCA1 is not functional, because BRCA1 promotes resection [30–32]. Since EEPD1 can operate independently of BRCA1 to initiate EXO1-mediated 5′ end resection after replication fork stalling [18, 19], this suggests that in the absence of functional BRCA1, EEPD1 can initiate the 5′ end resection needed for generation of the 3′ SS DNA required for RAD51 loading.

Repairing stressed replication forks is a high priority for the cell. If stressed replication forks are not repaired in timely manner, they may convert into toxic structures that make fork restart difficult [3, 9, 12, 13, 33], leading to mitotic catastrophe as demonstrated by nuclear abnormalities, including nuclear bridges and micronuclei. These nuclear abnormalities can arise from non-homologous end joining (NHEJ)-mediated fusion of free DS ends at unrepaired replication forks [3, 34–36]. Unbalanced chromosomal fusions can result in chromosomes without centromeres, which are retained as micronuclei after mitosis, and chromosomes with two centromeres, which form chromosomal bridges between daughter cells during mitosis [36, 37]. Since BRCA1/2 mutant cancer cells use RAD52 as an escape pathway for HR-mediated replication fork repair and restart, depleting RAD52 causes mitotic catastrophe and synthetic lethality in these cells. Thus, RAD52 has emerged as a target of interest for pharmaceutical intervention for novel synthetic lethal treatment strategies for BRCA1/2 mutant cancers [38–40]. However, the molecular mechanism by which RAD52 deficiency causes synthetic lethality of BRCA1/2 mutant cancer cells has not been identified [26, 27, 29].

In this study we show that EEPD1 is required for death of BRCA1 mutant breast cancer cells that have been depleted of RAD52. Specifically we show that depletion of EEPD1 rescues the synthetic lethality of RAD52-depleted, BRCA1 mutant breast cancer cells. Co-depletion of EEPD1 with RAD52 promotes restart of stalled replication forks, and suppresses chromosome aberrations and mitotic catastrophe compared to RAD52 depletion alone. These results suggest that EEPD1 may play a role in generating a toxic replication fork intermediate that leads to mitotic catastrophe.

## Methods

### Cell culture, transfection, and survival assays

EEPD1 and/or RAD52 were selectively depleted using small interfering RNA (siRNA) transient transfection (Lipofectamine RNAiMAX transfection Reagent, Life Technologies). SMARTpool ON-TARGETplus Non-target pool (SiRNA control) (D-001810-10-20), EEPD1 SiRNA (L-014641-01-0020), RAD52 SiRNA (L-011760-00-0010), X-ray repair cross-complementing protein (XRCC4) siRNA (L-004494-00-0005), DNA ligase IV (LIG4) (L-004254-00-0005), polymerase theta (POLQ) siRNA (L-015180-01-0010) and BRCA1 siRNA (L-003461-00-0005) were purchased from Dhamarcon RNAi Technologies (Pittsburgh, PA, USA). Briefly, the day prior to transfection, cancer cells (MDA-MB-436, SUM149PT and MCF7) were plated at a density of 1.4 × 10^5^ per well. Transfection reagents were prepared by mixing 6 μl of RNAiMAX/250 μl Opti-MEM (Life Technologies) to 50 nM of siRNA/250 μl Opti-MEM at room temperature (RT) for 20 min before adding to cells. Between 4 h and 6 h after transfection, 0.5 ml of fresh medium was added to each well for all cell types except MCF7. Instead of 50 nM siRNA, 10 nM of siRNA was used in all MCF7 transfections [27]. Cells were harvested at 2 days post-transfection for clonal colony formation (survival), western analysis, immunofluorescence or other assays. All experiments were performed at least three times in triplicate (n > 9).

Clonal survival was determined by seeding transfected cells (800 MDA-MB-436 (^+^ or ^-/-^), 1000 MCF7 or 1000 SUM149PT) per well of a 6-well plate and cells were allowed to expand for 10–12 days (MDA-MB-436 BRCA1^+^, SUM149PT) or 14–18 days (MDA-MB-436 BRCA1^-/-^, MCF7). Cells were then rinsed with 1 × PBS, fixed with 1% formaldehyde for 10 min and stained with 0.1% crystal violet before counting. Colonies with > 50 cells were counted as a surviving clone. For hydroxyurea (HU) treatment (H8627) (Sigma, St. Louis, MO, USA), cells were treated with 10 mM HU overnight at the 48-h post-transfection time point and subsequently harvested for colony formation assay as described. The unpaired Student *t* test was used for all statistical analysis, unless otherwise indicated.

MDA-MB-436 breast cancer cells (BRCA1 mutant (^-/-^) or replete (^+^)) (ATCC, Manassas, VA, USA) and MCF7 (ATCC) were cultured in D-MEM (Life Technologies, Carlsbad, CA, USA) supplemented with 10% fetal bovine serum (Hyclone) and 1% penicillin and streptomycin (Life Technologies).

SUM149PT BRCA1-/- breast cancer cells (Asterand Bioscience, Detroit, MI, USA) were cultured in Ham’s F-12 medium (Invitrogen) supplemented with 5% heat inactivated FBS (Hyclone), 10 mM HEPES (Invitrogen), 1 μg/ml hydrocortisone (Sigma) and 5 μg/ml insulin (Sigma).

### Western blot analysis

Protein expression of EEPD1, RAD52, DNA ligase 4 (LIG4), XRCC4, POLQ, BRCA1, BRCA2, and the constitutively expressed cyclophilin B was monitored by standard western blotting. EEPD1 expression was detected by a custom-produced mouse polyclonal antibody to EEPD1 protein (Interdisciplinary Center for Biotechnology Research Core Facility, UF, Gainesville, FL, USA) [19, 41]. RAD52, LIG4, BRCA1, and BRCA2 antibodies were purchased from Santa Cruz Biotech (sc-8350, sc-365341, sc-271299, sc-6954 and sc-1818). POLQ and XRCC4 antibodies were purchased from ThermoFisher Scientific (PA5-39885 and PA5-27104). Cyclophilin B antibodies were purchased from Abcam (ab178397) (Cambridge, MA, USA). Secondary antibodies used for enhanced chemiluminescence (EC) detection were ECL Rabbit IgG, HRP-linked Whole Ab (NA934-1ML), HRP-conjugated mouse secondary antibody (NA931-1ML) (Thermo Fisher Scientific, Waltham, MA, USA) and HRP-conjugated goat IgG (sc-2020, Santa Cruz Biotec). SuperSignal West Pico Chemiluminescent Substrate (ECL) (34078) and High Performance Chemiluminescence film; Amersham Hyperfilm ECL (45001508) were purchased from Thermo Fisher Scientific.

Expression levels of proteins involved in the ATM/ATR DNA damage signaling pathway were examined using ataxia-telangiectasia mutated kinase (ATM) (2873), p-ATM (5883), ATM-related and Rad3-related kinase (ATR) (2790), Checkpoint kinase 1 (Chk1) (2341), p-Chk1 (2348), Chk2 (2662) and p-Chk2 (2662) antibodies from Cell Signaling Technology (Danvers, MA, USA), p-ATR (GTX128145) antibodies from GeneTex (Irvine, CA, USA), replication protein A 32 (RPA32) (A300-244A) and p-RPA32 (A300-245A) antibodies from Bethyl Laboratories (Montgomery, TX, USA).

### Immunofluorescence

Immunofluorescence foci assays were performed as we previously described with minor modifications [19]. In brief, MDA-MB-436 BRCA1^-/-^ cells were cultured on coverslips followed by siRNA transfection. At the predetermined time points (1, 2, 3, or 4 days post transfection), cells were fixed with 1% formaldehyde for 10 min at ambient temperature, rinsed with 1 × PBS, incubated with methanol for > 5 min at − 20 °C, rinsed with 1 × PBS and permeabalized with 0.1% Triton-X for 3 min before incubation with phosphorylated histone 2A family member X (γH2AX) antibodies (05-636) (1:200) (Millipore, Temecula, CA, USA) at 4 °C overnight. The cells were then rinsed with 1 × PBS multiple times. Secondary antibodies (Goat anti-Mouse IgG, Alexa Fluor® 568 conjugate, A11004) (1:400) (Thermo Fisher) were added to the cells at ambient temperature and protected from light for 1 h. After washing thrice with 1 × PBS, coverslips were mounted in an anti-fade solution containing 4',6-diamidino-2-phenylindole (DAPI).

All samples were analyzed using either a Zeiss fluorescence microscope (Axiovert 200 M) (Carl Zeiss Microscopy, LLC, Thornwood, NY, USA) or a Leica TCS SP5 confocal scanning microscope (Leica Microsystems, Exton, PA, USA). Immunofluorescence images were taken using a Hamamatsu ORCA-ER digital camera (Hamamatsu Photonics K.K, Bridgewater, NJ, USA) and processed by Zeiss Axiovision Release 4.6 software. Images from confocal microscopy were processed by Leica LAS AF imaging software. Cells with ≥ 5 foci were scored as positive. Photomicrographs of distinct cell populations were taken at equal magnifications and equal fluorescence intensities. To assess nuclear structural abnormalities (micronuclei and post-mitotic bridging), MDA-MB-436 BRCA1^-/-^ cells, with or without EEPD1 and/or RAD52 depletion, were fixed as described above, and stained with 300 nM DAPI (Beckman) in PBS for 5 min. After washing with PBS, coverslips were mounted in anti-fade solution and analyzed using confocal microscopy. Each immunofluorescence assay was performed at least three times.

### Analysis of chromosome breaks

Cytogenetic analysis of chromosome breaks was performed as we described with minor modifications [19]. Briefly, 24 h prior to transfection, 1.6 × 10^5^ cells were plated into wells containing 1.5- mm coverslips. Cells were transfected with control, EEPD1, and/or RAD52 siRNA using Lipofectamine RNAiMAX reagent as described. After 48 h, cells were treated with Colcemid (final concentration of 0.1 ug/ml) (Sigma) for 2 h at 37 °C, 5% CO2. After 2 h, cells were treated with 75 mM KCl at 37 °C, 5% CO2. After 15 min, 0.1 volume of fixative (methanol/acetic acid 3:1) was added to the KCL for a few seconds and the supernatant was aspirated and replaced by 1 ml fresh fixative for 5 min at room temperature. The step was repeated two more times. Coverslips were then left for air drying and cells were rehydrated with PBS for 5 minutes and stained for the Giemsa Stain (Karyomax Giemsa Stain, 10092) (Gibco, Carlsbad, CA, USA) for 5 min at RT. The coverslips were rinsed three times with deionized water, mounted and examined with a × 63 objective connected to a Zeiss Axiovert 200 M microscope. At least 50 metaphases were analyzed for chromatid breaks in each cell preparation. Data were collected from three separate experiments.

### DNA fiber analysis of replication fork repair and restart

DNA fiber analysis for measuring stalled replication fork repair and restart was performed as we previously described [30, 31]. Briefly, 600,000 of MDA MB 436 BRCA1^-/-^ cells were incubated overnight at 37 °C in 6-well plates, then 20 mM iodo-deoxyuridine (IdU) was added to the growth medium and incubated for 20 min at 37 °C. The IdU medium was removed and cells washed in fresh medium. Cells were then treated with 5 mM HU for 120 min or mock-treated. The HU-containing medium was replaced with fresh medium containing 100 mM chloro-deoxyuridine (CldU). Cells were then incubated for varying times at 37 °C. The CidU medium was removed, cells harvested, resuspended in PBS, and ∼ 1000 cells were transferred to a positively charged microscope slide (Superfrost/Plus, Daigger), and processed for DNA fiber analysis as we described previously [32]. Slides were mounted in PermaFluor aqueous, self-sealing mounting medium (Thermo Scientific), and DNA fibers were visualized using an Olympus FV1000D confocal scanning microscope (Olympus America Inc., Center Valley, PA, USA). Images were analyzed using ImageJ software.

### DNA resection at stalled replication forks

Single-label DNA fiber end-resection analysis was carried out as previously described with some modifications [16, 42]. Briefly, MDA-MB-436 BRCA1 mutant cells transfected with the indicated siRNA were grown in 6-well dishes (2 × 10^5^ cells/well), and then 20 μM IdU was added to the growth medium and incubated for 45 min at 37 °C. After washing with fresh medium, cells were treated with 5 mM hydroxyurea for 0 or 10 h at 37 °C. Cells were harvested and suspended in PBS, and 1000 cells were transferred to a positively charged microscope slide and processed for DNA fiber analysis as we described previously [43]. Slides were mounted in PermaFluor aqueous, self-sealing mounting medium (Thermo Scientific), and DNA fibers were visualized using a confocal microscope (Olympus, FV1000D, × 63 oil immersion objective). Images were analyzed using ImageJ software.

## Results

### Depletion of EEPD1 promotes survival in BRCA1-mutated and RAD52-depleted breast cancer cells

**Fig. 1:**
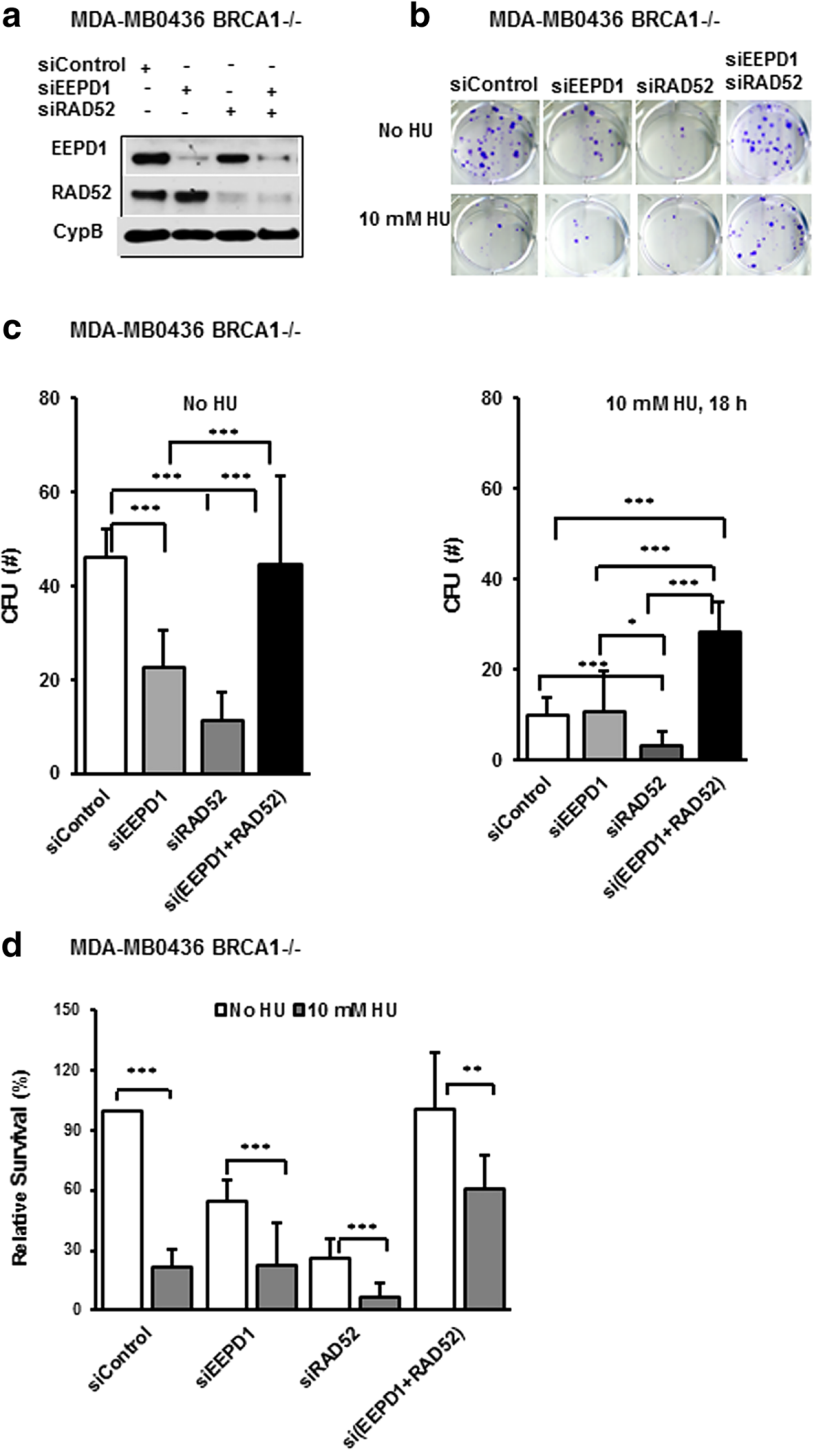
Depletion of endonuclease/exonuclease/phosphatase family domain-containing-1 (EEPD1) promotes survival in breast cancer susceptibility protein-1 (BRCA1)-mutated and radiation repair protein 52 (RAD52)-deficient breast cancer cells. MDA-MB-436 BRCA1^-/-^ cells were transfected with control, EEPD1 and/or RAD52 siRNA for 48 h and then cells were plated for colony formation unit (CFU) clonal survival assays. **a** Western blot analysis of EEPD1 and RAD52 depletion with cyclophilin B (CypB) as a loading control. **b** Representative images of clonal colony formation for each condition after 14 days. **c** Quantitative analysis of colony formation under unstressed (no hydroxyurea (HU)) or stress conditions (10 mM hydroxyurea (HU) overnight). **d** Relative survival data from **c** normalized by unstressed scrambled control siRNA. Each experiment was performed more than three distinct times in triplicate. For this and all subsequent figures, *p < 0.05, **p < 0.01, ***p < 0.001

MDA-MB-436 BRCA1^-/-^ breast cancer cells were depleted of EEPD1 and/or RAD52 using siRNA. Consistent with previous studies [26, 27], RAD52 depletion in BRCA1^-/-^ cells reduced cell viability threefold compared to control siRNA-transfected cells, as demonstrated by reduced colony formation units (CFUs) (Fig. 1a–d). RAD52 depletion did not affect clonal cell survival in MDA-MD-436 BRCA1^+^ breast cancer cells (Additional file 1: Figure S1A − C). When we depleted BRCA1 in MDA-MB-436 BRCA1^+^ cells, it re-sensitized the cells to RAD52 depletion (Additional file 1: Figure S1D–F). Depletion of EEPD1 in BRCA1^-/-^ cells also reduced clonal cell survival twofold, consistent with its known role in replication stress repair [19]. Interestingly, depletion of both RAD52 and EEPD1 together fully restored clonal cell viability to levels indistinguishable from control cells (Fig. 1a–c). We repeated the clonal survival experiments with a second BRCA1 mutant breast cancer cell line, SUM149PT [44]. When depleted of RAD52, synthetic lethality was observed in these BRCA1 mutant cells as well (Additional file 2: Figure S2). When EEPD1 and RAD52 were co-depleted, clonal cell viability was restored to levels that were comparable to control cells (Additional file 2: Figure S2).

Next, we created HR-deficient MCF7 breast cancer cells by transiently depleting BRCA1. We found that BRCA1-deficient MCF7 cells were highly sensitive to RAD52 depletion, consistent with a previous report (Additional file 3: Figure S3A–C) [27]. Interestingly, our data showed that, unlike BRCA1 mutant MDA-MB-436 and SUM149PT cancer cells, BRCA1-depleted MCF7 cancer cells were not sensitive to EEPD1 depletion (Fig. 1, Additional file 2: Figure S2 and Additional file 3: Figure S3D–F). However, co-depletion of EEPD1 and RAD52 did promote clonal cell viability in BRCA1-suppressed MCF7 cells (Additional file 3: Figure S3D–F). Thus, the cell death seen in BRCA1-deficient breast cancer cells with RAD52 depletion requires the HR endonuclease EEPD1.

We also examined survival of MDA-MB-436 BRCA1^-/-^ cancer cells after 18 h treatment with 10 mM HU (Fig. 1c, d), which causes replication fork stalling and collapse [11]. As expected, BRCA1^-/-^ breast cancer cells were sensitive to HU (control siRNA), and this was exacerbated by RAD52 depletion (Fig. 1c, d). EEPD1 depletion in BRCA1-proficient cells causes sensitivity to HU [19] and EEPD1 depletion in BRCA1^-/-^ cells also seemed to cause further sensitization to HU (Fig. 1c, d). Importantly, co-depletion of RAD52 and EEPD1 decreased HU sensitivity of BRCA1^-/-^ cells. In fact, the doubly depleted cells were significantly more resistant to HU than control cells (Fig. 1c, d).

### RAD52 depletion increases genome instability in BRCA1-deficient breast cancer cells

**Fig. 2:**
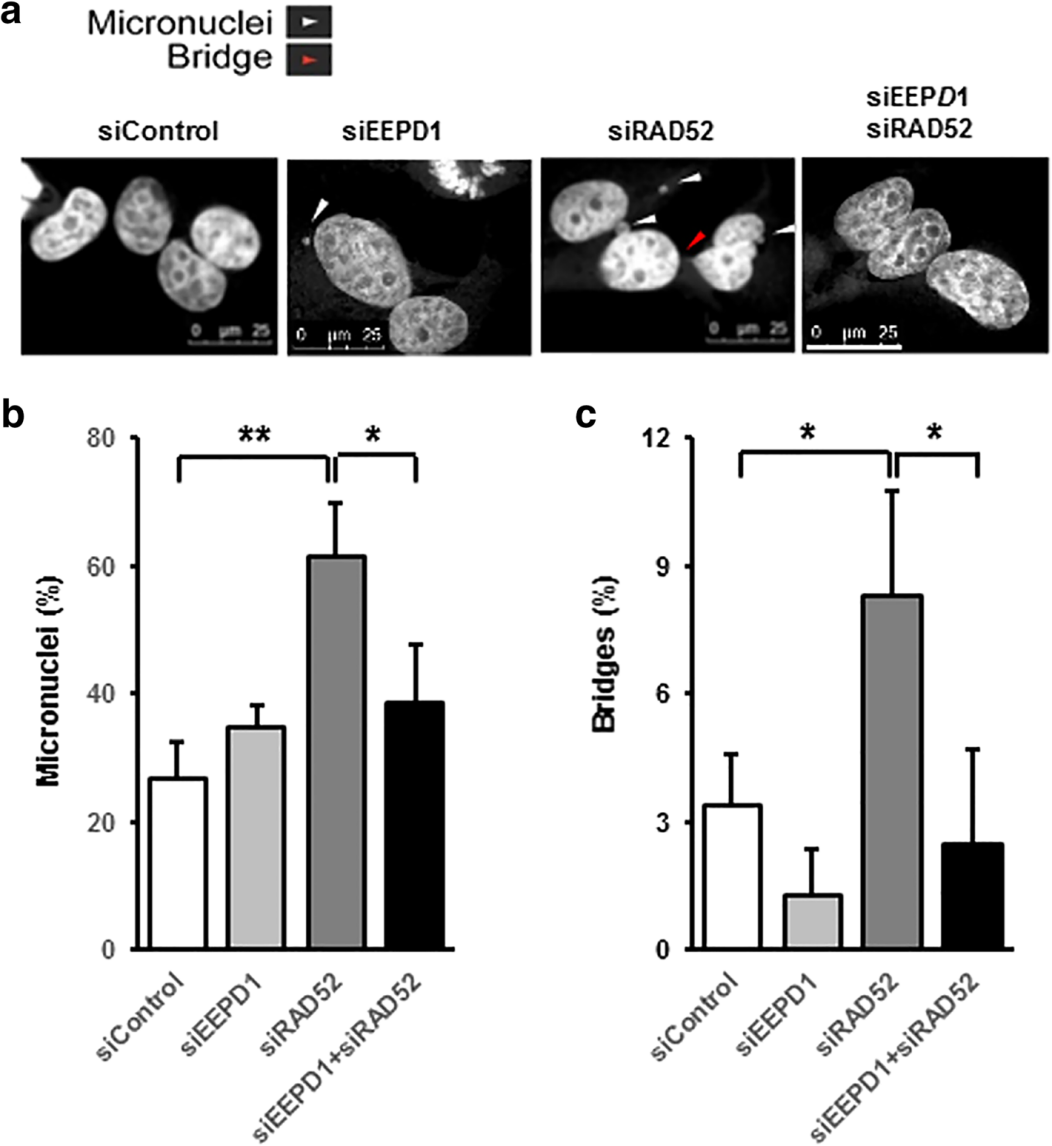
Restoration of genome stability by endonuclease/exonuclease/phosphatase family domain-containing-1 (EEPD1) depletion in radiation repair protein 52 (RAD52)-depleted breast cancer susceptibility protein-1 (BRCA1)-deficient breast cancer cells. MDA-MB-436 BRCA1^-/-^ cells were depleted of EEPD1 and/or RAD52, stained with 4',6-diamidino-2-phenylindole (DAPI) and analyzed for nuclear aberrations. **a** Representative photomicrographs. White arrowheads indicate micronuclei and red arrowhead indicates a nuclear bridge. Quantitative analysis of cell population that carried micronuclei (**b**) or nuclear bridges (**c**). Each data set was collected from five different × 40 fields from three distinct experiments. Mean ± SEM is shown. Scale = 25 uM

**Fig. 3:**
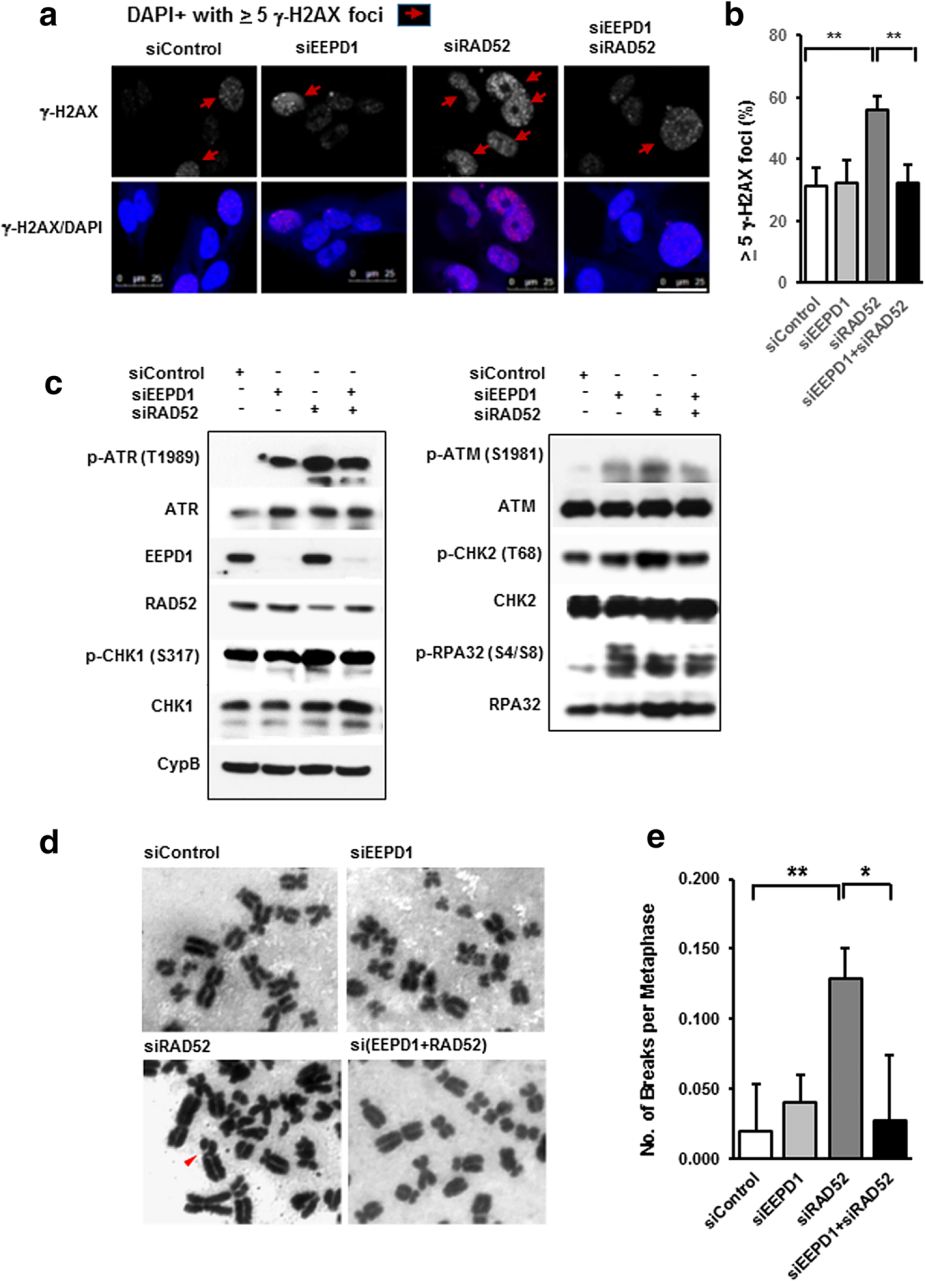
Genomic instability in radiation repair protein 52 (RAD52)-depleted breast cancer susceptibility protein-1 (BRCA1)-deficient breast cancer cells. **a** Representation photomicrographs of immunofluorescence staining of DNA double strand break marker, phosphorylated histone 2A family member X (γ-H2AX), showed various levels of nuclear foci formation in MDA- MB-436 BRCA1^-/-^ cells with or without endonuclease/exonuclease/phosphatase family domain-containing-1 (EEPD1) and/or RAD52 depletion. **b** Quantitative analysis of γ-H2Ax foci-positive (≥5 foci per cell) cell population in each condition. Cells were counterstained with 4',6-diamidino-2-phenylindole (DAPI). Each set of data was collected from five different × 40 fields from three distinct experiments. Mean + SEM is shown. Scale = 25 uM. **c** Western blot analysis of EEPD1, RAD52, phosphorylated ataxia-telangiectasia mutated kinase (ATM) and Rad3-related kinase (p-ATR), ATR (total), phosphorylated checkpoint kinase 1 (p-CHK1), CHK1 (total), p-ATM (phosphorylated), ATM (total), p-CHK2 (phosphorylated), CHK2 (total), phosphorylated p-replication protein A 32 kDa subunit (p-RPA32), RPA32 (total) and cyclophilin B (CypB) as a loading control in EEPD1and/or RAD52 depleted cells. **d** Cytogenetics showing an increase in chromosome breakage after RAD52 depletion in BRCA1^-/-^ cells. Representative images of metaphases from each condition. Arrowhead indicates sites of chromosome breakage. **e** Quantitation of chromosomal breakage in each condition, with mean ± SEM shown (three distinct experiments with > 20 metaphases counted per experiment)

Previous studies found that RAD52 depletion in BRCA2-defective breast cancer cells is associated with spontaneous and radiation-induced chromosomal aberrations [26]. We depleted RAD52 in MDA-MB-436 BRCA1^-/-^ breast cancer cells, stained nuclei with DAPI and measured nuclear abnormalities. We assessed retained chromosomes, seen as micronuclei, and mis-segregated chromosomes, seen as post-mitotic bridges [36, 37]. We found that a significantly higher fraction of RAD52-depleted BRCA1 mutant cells had micronuclei compared to control cells (Fig. 2a, b). RAD52 depletion also significantly increased the frequency of bridges in the BRCA1 mutant cells (Fig. 2a, c). Chromosomal breaks are specifically associated with unrepaired replication stress [45], and can be quantified using metaphase analysis. Metaphase analysis also showed a marked increase in chromosomal breaks when RAD52 was depleted in BRCA1 mutant cells (Fig. 3d, e).

The synthetic lethality of combined RAD52 and BRCA1/2 deficiency is thought to reflect inefficient repair and restart of stressed replication forks, resulting in their collapse [26, 27]. These collapsed forks are marked by γ-H2AX, reflecting chromatin changes adjacent to the unrepaired DS ends seen in collapsed forks [11]. Consistent with this model, depletion of RAD52 in BRCA1-deficient breast cancer cells increased γ-H2AX nuclear foci compared to control cells (Fig. 3a, b). The DNA damage signaling kinases ATR and ATM are activated by phosphorylation in the DNA damage response [46]. Analysis of phosphorylated ATR and ATM proteins and their downstream signal transducers, phosphorylated CHK1 and CHK2, confirmed that these DNA damage signaling pathways were indeed highly activated in RAD52-depleted BRCA1^-^ cells (Fig. 3c), consistent with replication fork collapse.

### EEPD1 depletion restores genome stability in RAD52-depleted BRCA1 mutant breast cancer cells

When EEPD1 was co-depleted with RAD52 in BRCA1^-/-^ breast cancer cells, levels of micronuclei and nuclear bridges were reduced to levels comparable to those seen in control cells (Fig. 2b, c). In addition, compared to RAD52 depletion alone, double depletion of EEPD1 and RAD52 resulted in fewer γ-H2AX-positive cells (Fig. 3a). Metaphase analysis also found that co-depleting EEPD1 with RAD52 reduced chromosome breaks in BRCA1 mutant cells back to control levels (Figs. 2 and 3). For each of the genome instability endpoints (nuclear abnormalities, chromosome breaks, and γ-H2AX-positive cells), levels in BRCA1-deficient cells with EEPD1/RAD52 double depletion were comparable to levels of control cells. Together, these results indicate that the genome instability caused by depleting RAD52 in BRCA1^-/-^ cells is dependent on the HR endonuclease EEPD1.

### EEPD1 depletion rescues stressed replication forks in RAD52-depleted BRCA1-deficient breast cancer cells

**Fig. 4:**
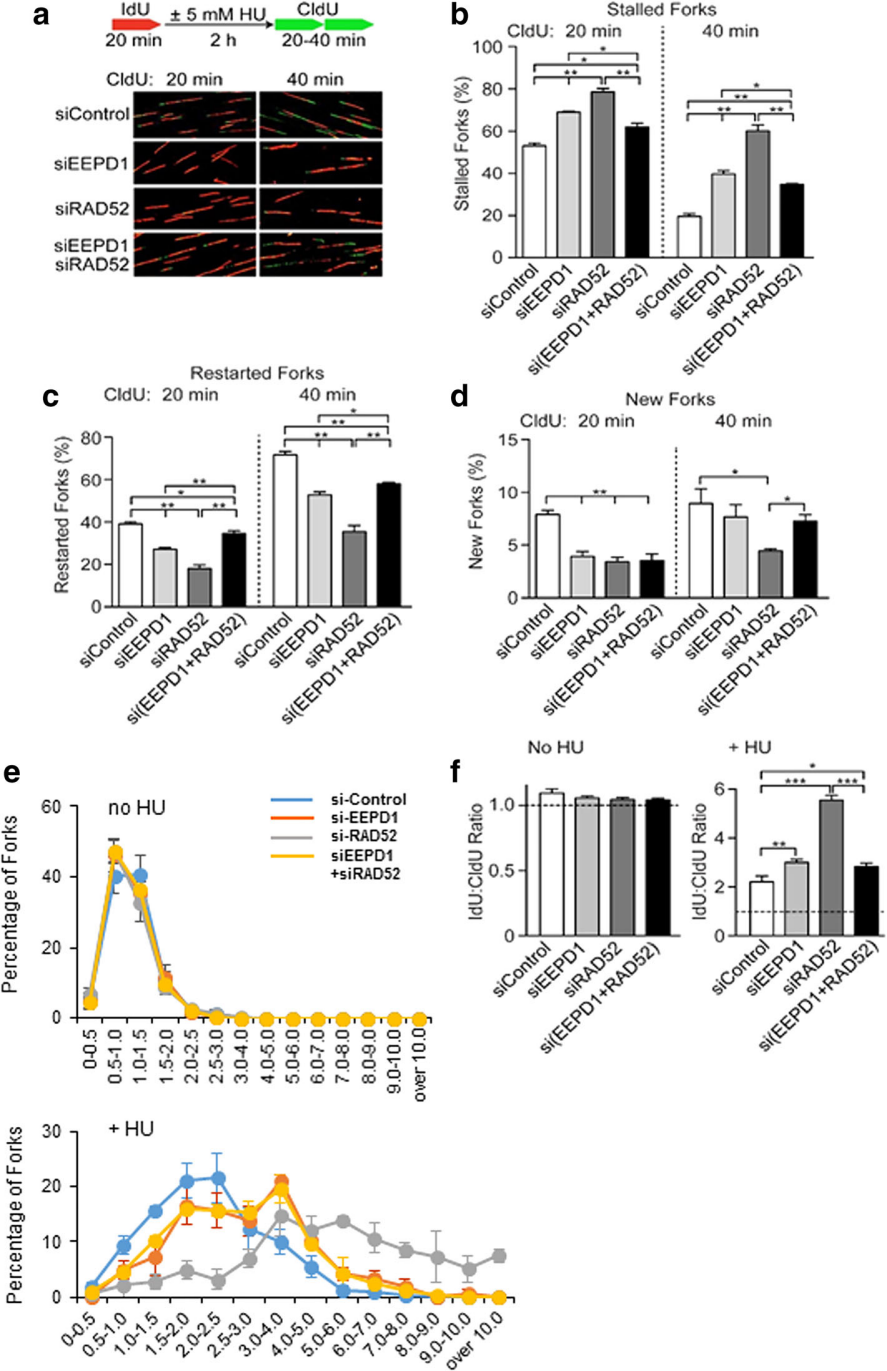
Endonuclease/exonuclease/phosphatase family domain-containing-1 (EEPD1) depletion rescues stressed replication forks in radiation repair protein 52 (RAD52)-depleted breast cancer susceptibility protein-1 (BRCA1)-deficient breast cancer cells. DNA fiber analysis of stalled, restarted or new forks in MDA-MB-436 BRCA1^-/-^ cells transfected with control, EEPD1 and/or RAD52 siRNA. **a** Schematic diagram depicts steps for the DNA fiber assay and representative images of DNA fibers from each condition. Forks prior to stress with hydroxyurea (HU) are stained red with iodo-deoxyuridine (IdU), new forks after relieving stress are stained green with chloro-deoxyuridine (CldU). Quantitative analysis of stalled (only red fibers) (**b**), restarted (red then green fibers) (**c**) and new forks (only green fibers) (**d**) after release from HU replication stress. e, f Quantitative analysis of stalled versus restarted replication forks (IdU:CIdU) ratios under unstressed condition or stressed condition (5 mM HU for 120 min). Three distinct experiments per condition (>121 fibers measured per condition for each experiment)

We next used DNA fiber analysis to measure repair and restart of stressed replication forks in either mock-treated or HU-treated cells for 20 or 40 min after release from HU (Fig. 4a). DNA fibers were analyzed for unrepaired forks (red IdU label only), repaired and restarted forks (both red IdU and green CldU label on a single fiber), and newly originated forks (green CIdU label only). As shown in Fig. 4b–d, depletion of RAD52 in BRCA1^-/-^ cells increases the frequency of forks that fail to restart, and this was apparent with 20 or 40 min recovery from HU replication stress. Depletion of EEPD1 also reduced fork restart, as reported previously [19], albeit to a lesser degree than RAD52 depletion in the BRCA1^-/-^ cancer cells. Double depletion of RAD52 and EEPD1 partially restored fork restart to a level between control and RAD52 depletion alone during the 20 and 40 min recovery periods.

When a replication fork stalls, replication recovery can result from fork restart, or more rarely from activation of nearby dormant replication origins [11, 19, 47]. RAD52 and/or EEPD1 depletion reduced new replication fork firing at 20 min recovery after HU replication stress (Fig. 4d). After 40 min of recovery from HU removal, a significant decrease in new forks was only seen with RAD52 depletion alone, thus co-depletion of EEPD1 suppressed the effect of RAD52 depletion on new fork initiation. Thus, RAD52 depletion in BRCA1^-/-^ cells suppresses both fork repair and restart and new fork initiation, and these effects are suppressed by co-depletion of EEPD1.

We also measured IdU:CldU tract-length ratios, which provides quantitative information about the extent of replication recovery at forks that restarted. Under non-stress conditions, IdU and CldU track lengths are approximately equal, giving an IdU:CldU ration of ~ 1.0, which means that the stressed replication fork fully recovered to its baseline progression rate. As shown in the top graph of Fig. 4e, unstressed BRCA1^-/-^ cells displayed a narrow distribution of IdU:CldU ratios centered around 1.0, and this was unaffected by depletion of RAD52 and/or EEPD1 (Fig. 4f, left panel). In cells treated with HU between IdU and CldU pulses, broader distributions with higher IdU:CldU ratios are observed (Fig. 4e, bottom graph). Higher IdU:CIdU ratios reflect inefficient replication recovery among stressed forks. In this assay, inefficient replication fork recovery may reflect slower fork repair and/or slower progression upon restart. Under HU stress conditions, EEPD1 depletion slightly increased the average IdU:CldU ratio, whereas RAD52 depletion increased the ratio by >2-fold (Fig. 4f, right panel). Interestingly, the ratio with double depletion of EEPD1 and RAD52 was similar to that with EEPD1 depletion alone, indicating that the defect in replication recovery caused by RAD52 depletion in BRCA1-defective cells is largely suppressed by co-depletion of EEPD1.

Stressed replication fork degradation can be measured by single-label DNA fiber analysis [16, 42, 43]. Such degradation of recently labeled DNA can be from normal end resection initiating HR or from nuclease destruction of collapsed replication forks [48–53]. We have previously demonstrated that EEPD1 is essential for stressed fork degradation from end resection in this assay [18, 41]. When we measured HU-stressed replication fork degradation in BRCA1 mutant breast cancer cells we found that depleting RAD52 or EEPD1 led to less stressed replication fork degradation, but co-depletion of both restored degradation to control levels (Additional file 4: Figure S4). While depleting EEPD1 would result in decreased end resection, it is more likely that RAD52 depletion results in stressed fork nuclease destruction [23, 26, 27].

### BRCA1-deficient breast cancer cells depleted of both EEPD1 and RAD52 rely on alternative non-homologous end-joining pathway (aNHEJ) for survival

**Fig. 5:**
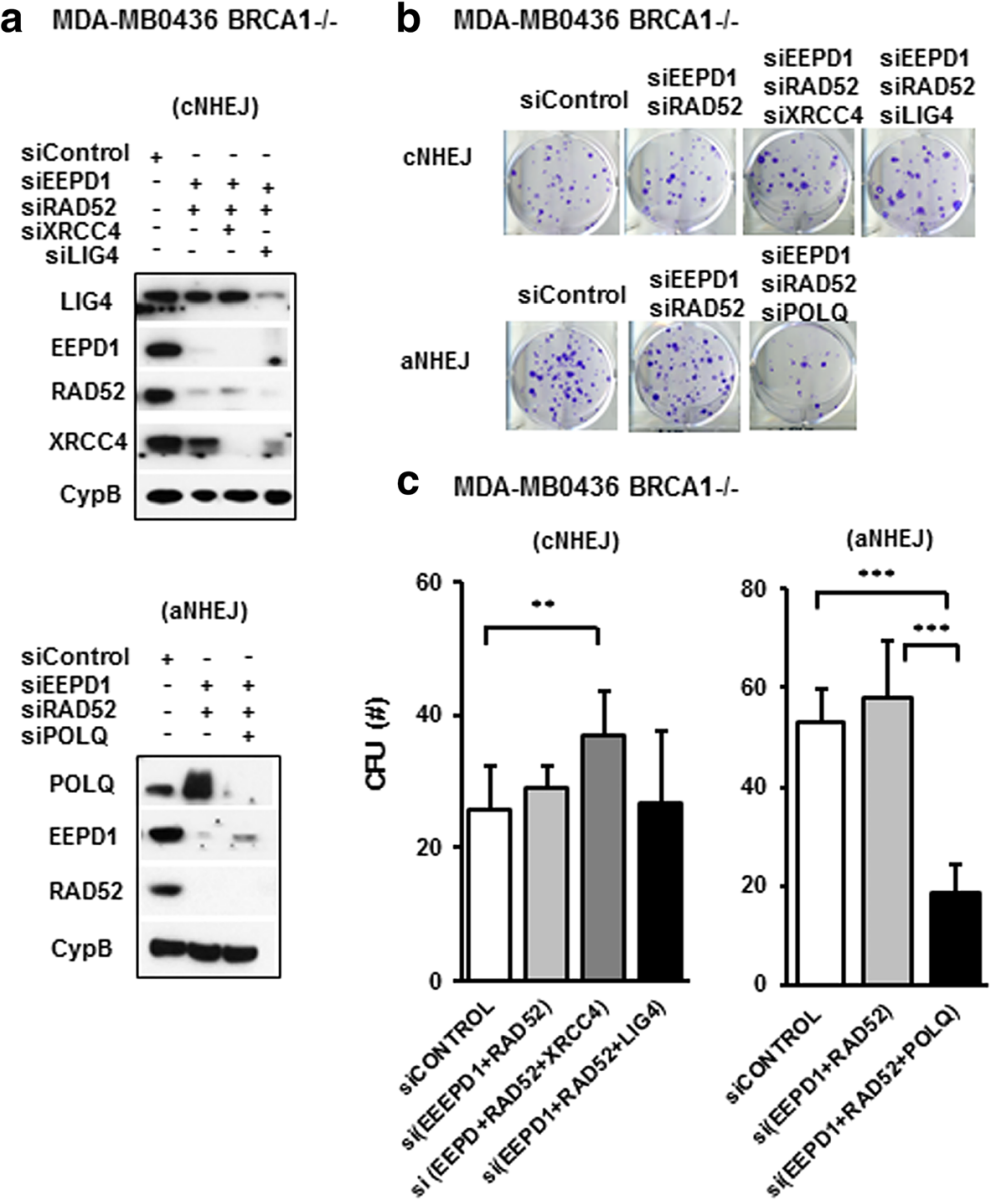
Breast cancer susceptibility protein-1 (BRCA1)-deficient breast cancer cells depleted of both EEPD1 and RAD52 rely on alternative non-homologous end-joining pathway (aNHEJ) for survival. MDA-MB-436 BRCA1^-/-^ cells were transfected with control, endonuclease/exonuclease/phosphatase family domain-containing-1 (EEPD1)/radiation repair protein 52 (RAD52) with or without X-ray repair cross-complementing protein (XRCC4), DNA ligase IV (LIG4) or polymerase theta (POLQ) siRNA for 48 h and cells were plated for colony formation survival assays. **a** Western blot analysis of EEPD1, RAD52, XRCC4, LIG4, and POLQ protein expression with cyclophilin B (CypB) as a loading control in the transfected BRCA1^-/-^ cancer cells. **b** Representative images of clonal colonies from each condition after 14 days. **c** Quantitative analysis of colony formation that targeted the classical non-homologous end-joining (cNHEJ) pathway or aNHEJ pathway. Each experiment was performed more than three distinct times in triplicate (*p < 0.05, **p < 0.01, ***p < 0.001)

Our data confirm that synthetic lethality can be induced in BRCA1-deficient breast cancer cells through depletion of RAD52, possibly through the accumulation of a toxic fork repair intermediate. However, when EEPD1 is also depleted in the RAD52-depleted BRCA1-deficient breast cancer cells, the synthetic lethality is completely abolished. To address which escape pathway these EEPD1/RAD52 double-depletion BRCA1-deficient cells rely on for survival, we performed clonal cell survival assays with additional depletion of either X-ray repair cross-complementing protein (XRCC4) or DNA ligase IV (LIG4) to examine the classical NHEJ (cNHEJ)  pathway and POLQ to examine the alternative NHEJ pathway (aNHEJ) (Fig. 5).

When XRCC4 or LIG4 were depleted in the EEPD1/RAD52 co-depleted BRCA1-deficient breast cancer cells, no significant reduction in cell survival was observed (Fig. 5c). In addition, an increase of ~ 30% in clonal cell survival was observed in the EEPD1/RAD52/XRCC4 triple-depleted breast cancer cells compared to the EEPD1/RAD52 co-depletion cells respectively (Fig. 5c). This implies that the double-depleted EEPD1/RAD52 BRCA1 mutant cells do not rely on the cNHEJ pathway for survival. We then investigated whether aNHEJ was the escape pathway utilized by the EEPD1/RAD52 co-depleted BRCA1-deficient cancer cells for survival. We depleted POLQ, a DNA polymerase that promotes aNHEJ [46, 54], in the EEPD1/RAD52 co-depleted BRCA1-deficient cells. There was a reduction in cell survival in the EEPD1/RAD52/POLQ triple-depleted cells when compared to EEPD1/RAD52 co-depleted cells (Fig. 5c). This implies that the EEPD1/RAD52 co-depleted cells use the aNHEJ pathway to repair their stressed replication forks [55, 56].

We also repeated the cell survival assay with single depletion of XRCC4, LIG4 or POLQ in the MDA-MB-436 BRCA1-/- cells for comparison (Additional file 5: Figure S5). Little impact on cell survival was observed from XRCC4 or LIG4 singly depleted cells (Additional file 5: Figure S5), which was similar to the triple-depleted cells (XRCC4/EEPD1/RAD52 or LIG4/EEPD1/RAD52) (Fig. 5). When POLQ was depleted in the MDA-MB-436 BRCA1-/- cells, the level of synthetic lethality was also comparable to the triple-depleted cells (POLQ/EEPD1/RAD52) (Additional file 5: Figure S5 and Fig. 5).

## Discussion

BRCA1 mutant or BRCA2 mutant malignancies rely on RAD52 to repair stressed replication forks [26, 27, 29]. Depletion of RAD52 results in synthetic lethality of these homologous recombination (HR)-deficient cancers [26–29]. This study demonstrates that the synthetic lethality seen when RAD52 is depleted in BRCA1 mutant breast cancer cells depends on the HR endonuclease EEPD1. Previously, we have shown that EEPD1 nicks stressed replication forks to initiate 5′ end resection, which creates 3′ SS DNA for RAD51 loading to affect HR repair of stalled forks [18, 19]. The fact that synthetic lethality of RAD52-depleted BRCA1-deficient cells can be suppressed by downregulating EEPD1 implies that EEPD1 cleavage of stalled forks may create a toxic fork repair intermediate that is lethal if repair is not completed. In cells that lack BRCA1/2 and RAD52, EEPD1 would cleave stressed replication forks to permit 5′ end resection, but those cells would not progress past this repair intermediate. Repair would be arrested before RAD51-dependent homology-mediated single-strand invasion could occur. These cleaved replication forks would have free DS ends that could produce chromosomal fusions mediated by tumor protein P53 binding protein (53BP1)-dependent NHEJ [34, 35]. In BRCA1 mutant cancer cells, unrepaired replication forks can generate chromosomal fusions, leading to chromosomal instability and mitotic catastrophe [34–37]. Depleting 53BP1 rescues replication stress-induced chromosomal instability in these cells [19, 34, 35]. Thus, one mechanism of cell death in the RAD52-depleted BRCA1 mutant cells could be chromosomal fusion of cleaved, but unrepaired forks, resulting in mitotic catastrophe.

Interestingly, our study finds that depletion of XRCC4 or DNA ligase IV, key components in the cNHEJ repair pathway, has little impact on cell viability in the MDA-MB-436 BRCA1^-/-^ cells (Fig. 5, Additional file 5: Figure S5). In contrast, depletion of POLQ, a mediator of aNHEJ, induces severe synthetic lethality in the BRCA1^-/-^ cells (Fig. 5, Additional file 5: Figure S5). These observations are consistent with a previous report showing that BRCA1-deficient cancer cells are dependent on the aNHEJ pathway for replication [46]. Our study shows that MDA-MB-436 BRCA1^-/-^ cells rely heavily for survival upon the RAD52 recombination repair pathway, or if that fails, on the aNHEJ pathway (Fig. 1, Additional file 5: Figure S5). This implies that the RAD52 alternative HR repair pathway is effectively arrested in the co-depleted BRCA1 mutant cells, whereas the aNHEJ repair pathway remains active. In addition, unlike their ability to restore chromosomal integrity and replication efficiency (Figs. 2, 3 and 4), co-depletion of EEPD1 and RAD52 in BRCA1^-/-^ cells only has a moderate effect on DNA end-resection (Additional file 5: Figure S5). This is not surprising, since EEPD1 depletion would arrest replication fork repair before 5′ end resection, and repair of the fork might default to aNHEJ. POLQ-mediated aNHEJ typically does not compete with HR repair of stressed replication forks, probably because it is non-conservative, and the cell would designate it as a backup for conservative HR repair to protect its genome [55–57].

Repairing and restarting stressed replication forks is one of the highest priorities for any cell. Indeed, there is a great deal of evidence that cells will bypass DNA lesions to maintain replication rates [41, 47]. We and others have found that even delaying fork repair by 15–30 min can have lethal consequences [17, 19, 35, 47]. The data presented here suggest a model in which an EEPD1-cleaved replication fork repair intermediate is rapidly recognized by the cell as toxic, and in the absence of BRCA1 and RAD52, the free DS ends are shunted toward the aNHEJ repair pathway. Without the toxic EEPD1-cleaved fork repair intermediate, aNHEJ can repair the stressed replication fork and permit the RAD51-depleted BRCA1 mutant cell to survive [55–57]. This implies a hierarchy of replication fork repair pathways, with classical HR as favored, but in the absence of BRCA1/2, the cell chooses the RAD52 backup HR pathway [26–29]. When cells lack both classical HR and the RAD52-mediated backup pathway, they choose aNHEJ to repair stalled replication forks [13, 14, 55–57]. This may also explain why depleting both EEPD1 and RAD52 improves the survival of BRCA1 mutant cells after HU replication stress (Fig. 1c, d); this would force cells away from the RAD52 pathway to the more efficient but less conservative aNHEJ pathway. We had previously shown that EEPD1 actively represses aNHEJ while promoting HR, consistent with this hypothesis [41].

There are several other lines of evidence that endonucleases mediate stressed replication fork collapse and cell death if fork repair is not completed in a timely manner. For example, BRCA2 protects stressed replication forks from degradation by the nuclease Mre11 [41], perhaps by promoting timely loading of RAD51 onto end resected 3′ SS DNA [55, 56]. In BRCA2 mutant cells, the histone H3 lysine 4 methylase (MLL3/4) complex component Pax transcription activation domain interaction protein 1-like protein (PTIP) was found to recruit double-strand break repair nuclease (Mre11) to stalled replication forks, causing degradation of nascent DNA strands [57]. The Werner’s syndrome helicase (WRN) also stabilizes stressed replication forks and prevents their destruction by Mre11 [48]. WRN interacting protein1 (WRNIP1) stabilizes RAD51 on 3′ end-resected SS DNA, and prevents prolonged and excessive end resection by Mre11, thereby protecting stressed replication forks from degradation [49]. Finally, bi-orientation defect 1-like (BOD1L), a large protein with N-terminal homology to the mitotic regulator BOD1, is recruited to stressed replication forks where it stabilizes RAD51 on the 3′ SS end-resected DNA, which blocks further Bloom syndrome recQ-like helicase (BLM) unwinding and DNA2-mediated end resection [50]. In each of these examples, excessive nuclease degradation of stressed replication forks is prevented by proteins recruited to stressed forks to regulate end resection. These reports demonstrating that sophisticated mechanisms have evolved to protect excessive degradation of stressed forks provides further evidence that such repair intermediates are toxic to the cell.

There is a significant effort to create small molecular inhibitors of RAD52 in order to clinically treat BRCA1 and BRCA2 mutant breast and ovarian cancers [26, 38–40, 51]. Inhibition of RAD52 might be epistatic with PARP1 inhibition, since both strategies rely on the failure of replication fork repair and restart for their lethal effects, albeit at distinct steps in that pathway. Thus, combining PARP1 and RAD52 inhibitors to treat BRCA1 or BRCA2 mutant cancers might provide little or no therapeutic gain, and might increase normal tissue toxicity. Synthetic lethality from either PARP1 or RAD52 inhibition involves toxic replication fork repair intermediates that generate mitotic catastrophe and cell death [26, 29, 52, 53]. Given that depleting EEPD1 prevents synthetic lethality of RAD52 repression in BRCA1 mutant cancer cells, potential mechanisms by which cancer cells could become resistant to clinically useful RAD52 inhibitors are by repressing EEPD1 expression or acquiring EEPD1 loss-of-function mutations [52]. Either of these mechanisms would shunt stressed replication fork repair to aNHEJ [55–57]. Thus, EEPD1 could be used as a biomarker for treatments that target RAD52 in BRCA1/2 mutant cancers.

## Conclusion

The mechanism by which RAD52 depletion causes synthetic lethality in BRCA1 mutant cancer cells depends on the 5′ endonuclease EEPD1, which normally functions to cleave stressed replication forks to initiate HR repair. Such cells face death because these cleaved forks cannot complete repair when both BRCA1/2-mediated HR and the RAD52 backup pathway are impaired.

## Additional files

Additional file 1: Figure S1. RAD52 depletion does not induce synthetic lethality in BRCA1-replete breast cancer cells. **a**–**c** MDA-MB-436 cells with intact BRCA1 transduced back were transfected with control or RAD52 siRNA for 48 h and then cells were plated for colony formation survival assays. **a** Western blot analysis of RAD52 depletion. **b** Representation images of CFUs from each condition after 12 days. **c** Quantitative analysis of colony formation. (**d**–**f**) MDA-MB-436 BRCA1^+^ cells were transiently transfected with control or RAD52 siRNA, with or without BRCA1 siRNA, for 48 h. Cells were plated for colony formation survival assays. **d** Western blot analysis of RAD52 and BRCA1 depletion. **e** Representation images of CFUs from each condition after 12 days. **f** Quantitative analysis of colony formation. Each experiment was performed ≥ 3 distinct times in triplicate. (PDF 456 kb)

Additional file 2: Figure S2. EEPD1 depletion in SUM149PT BRCA1 mutant breast cancer cells rescues synthetic lethality from RAD52 depletion. **a**–**c** SUM149PT BRCA1^-/-^ cells were transiently transfected with control or RAD52 siRNA for 48 h and cells were plated for colony formation survival assays. **a** Western blot analysis of RAD52 and EEPD1 depletion. **b** Representation images of CFUs from each condition after 12 d. **c** Quantitative analysis of colony formation. Each experiment was performed ≥ 3 distinct times in triplicate. (PDF 263 kb)

Additional file 3: Figure S3. EEPD1 depletion in BRCA1-depleted MCF7 breast cancer cells rescues synthetic lethality from RAD52 depletion. **a**–**c** MCF7 BRCA1-proficient cells were transiently transfected with control or RAD52 siRNA, with or without BRCA1 siRNA, for 48 h. Cells were plated for colony formation survival assays. **a** Western blot analysis. **b** Representation images of CFUs from each condition after 14 days. **c** Quantitative analysis of colony formation. **d**–**f** MCF7 BRCA1-proficient cells were transiently transfected with control, EEPD1 and/or RAD52 siRNA, with BRCA1 siRNA, for 48 h. Cells were plated for colony formation survival assays. **d** Western blot analysis. **e** Representation images of CFUs from each condition after 14 days. **f** Quantitative analysis of colony formation. Each experiment was performed ≥ 3 distinct times in triplicate. (PDF 459 kb)

Additional file 4: Figure S4. Single-label DNA fiber analysis of stressed replication fork degradation. MDA-MB-436 BRCA1^-/-^ cells were transiently transfected with control, EEPD1 and/or RAD52 siRNA for 48 h and labeled with IdU for 45 min before proceeding to either 0 h or 10 h incubation with 5 mM HU. DNA degradation at stalled nascent replication forks was measured by fiber analysis. **a** Schematic diagram depicts steps for the DNA fiber assay and representative images of DNA fibers from each condition. IdU stained red (stalled forks). Quantitative analysis of IdU track length frequency at unstressed (no HU) (**b**), or HU-treated DNA fibers (**c**) from each condition. **d** Bar chart from the HU-treated IdU track length frequencies analysis. **c** and d are the same data. Co-depletion of both RAD52 and EEPD1 restores stressed replication fork degradation back to control levels. Three distinct experiments per condition (>100 fibers measured per condition for each experiment). (PDF 419 kb)

Additional file 5: Figure S5. cNHEJ DNA repair pathway is nonessential for MDA-MB-436 BRCA1 mutant breast cancer cells to survive. **a**–**c** MDA-MB-436 BRCA1^-/-^ cells were transfected with control or XRCC4 siRNA for 48 h and cells were plated for colony formation survival assays. **a** Western blot analysis of XRCC4 depletion. **b** Representation images of CFUs from each condition after 14 days. **c** Quantitative analysis of colony formation. **d**–**f** MDA-MB-436 BRCA1^-/-^ cells were transfected with control or LIG4 siRNA for 48 h and cells were plated for colony formation survival assays. **d** Western blot analysis of XRCC4 depletion. **e** Representation images of CFUs from each condition after 14 days. **f** Quantitative analysis of colony formation. **g**–**i** MDA-MB-436 BRCA1^-/-^ cells were transfected with control or POLQ siRNA for 48 h and cells were plated for colony formation survival assays. **g** Western blot analysis of POLQ depletion. **h** Representation images of CFUs from each condition after 14 days. **i** Quantitative analysis of colony formation. Each experiment was performed ≥ 3 distinct times in triplicate. (PDF 580 kb)

## Abbreviations

53BP1: Tumor protein P53 binding protein 1
aNHEJ: Alternative non-homologous end-joining
ATM: Ataxia-telangiectasia mutated kinase
ATR: ATM- and Rad3-related kinase
BLM: Bloom syndrome RecQ-like helicase
BOD1L: Bi-orientation defect 1-like protein
BRCA1: Breast cancer susceptibility protein-1
BRCA2: Breast cancer susceptibility protein-2
CHK1: Checkpoint kinase 1
CHK2: Checkpoint kinase 2
CIdU: Chloro-deoxyuridine
cNHEJ: Classical non-homologous end-joining
CtIP: C-terminal-binding protein 1
CypB: Cyclophilin B
Dna2: DNA replication helicase/nuclease 2
DS: Double-strand
DSBs: DNA Double-strand breaks
ECL: Enhanced chemiluminescence
EEPD1: Endonuclease/exonuclease/phosphatase family domain-containing-1
GEN1: Holliday junction 5′ flap endonuclease
HR: Homologous recombination
HRP: Horseradish phosphatase
HU: Hydroxyurea
IdU: Iodo-deoxyuridine
LIG4: DNA ligase IV
MLL3/4: Histone H3 lysine 4 methylase complexes
Mre11: Double-strand break repair nuclease
MUS81: MUS81 structure-specific endonuclease subunit
NHEJ: non-homologous end-joining
PALB2: Partner and localizer of BRCA2
PARP1: Poly(ADP-ribose) polymerase 1
PBS: Phosphate-buffered saline
POLQ: Polymerase theta
PTIP: Pax transcription activation domain interaction protein 1-like protein
RAD51: Radiation repair protein 51 recombinase
RAD52: Radiation repair protein 52
RIF1: Replication timing regulatory factor 1
RPA32: Replication protein A 32 kDa subunit
RT: Room temperature
SLX4: SLX4 Structure-specific endonuclease subunit
SS: Single-strand
WRN: The Werner’s syndrome helicase
WRN1P1: WRN interacting protein1
XRCC4: X-ray repair cross-complementing protein
γH2AX: Phosphorylated histone 2A family member X
