# Supplementary figures and images for: The endonuclease EEPD1 mediates synthetic lethality in RAD52-depleted BRCA1 mutant breast cancer cells
Source: Breast Cancer Res. 2017 Nov 16;19:122. doi: 10.1186/s13058-017-0912-8 (PMC5693420; doi:10.1186/s13058-017-0912-8)

# S Fig. 1

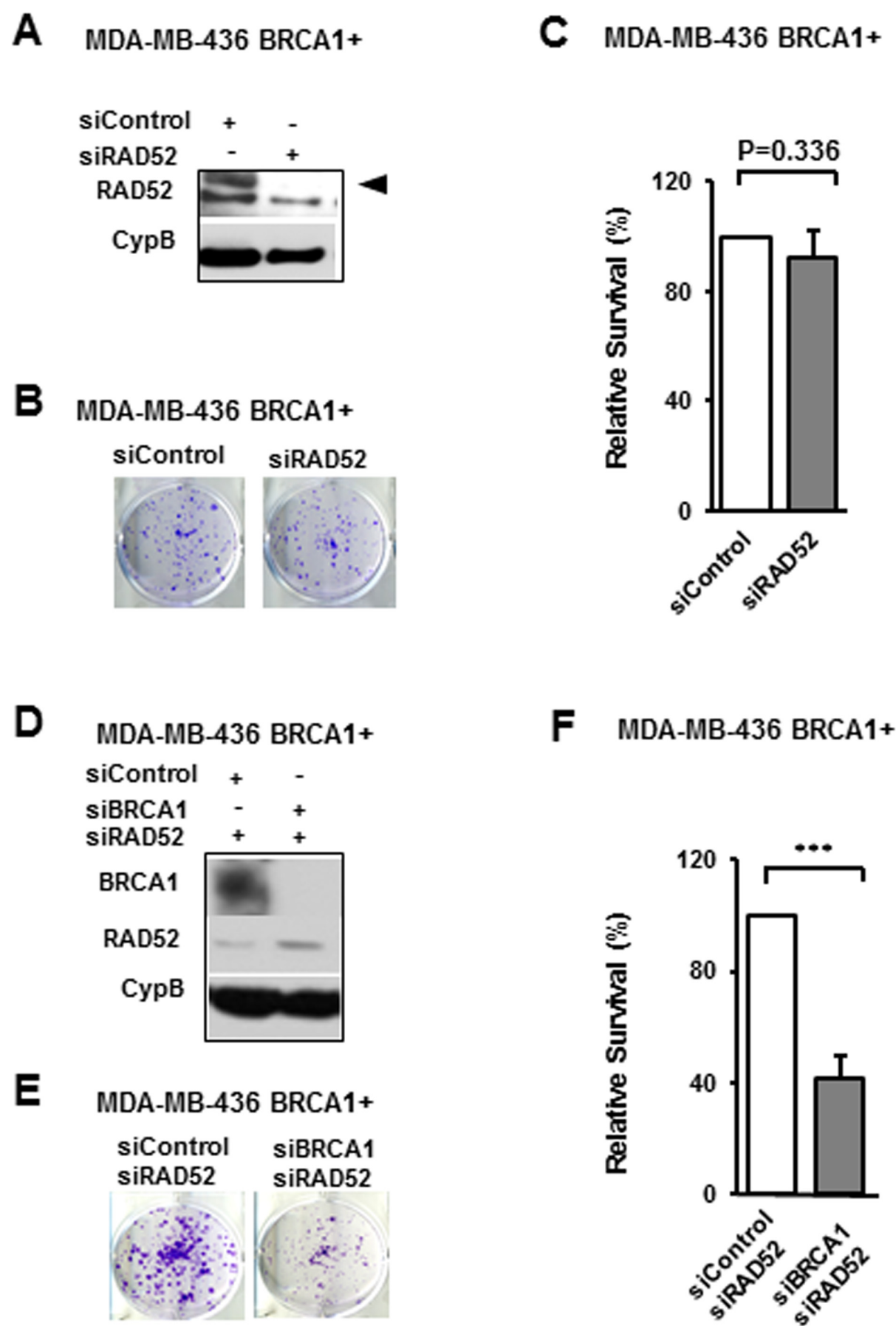

Supplement: Supplementary file 1 — RAD52 depletion does not induce synthetic lethality in BRCA1-replete breast cancer cells. a–c MDA-MB-436 cells with intact BRCA1 transduced back were transfected with control or RAD52 siRNA for 48 h and then cells were plated for colony formation survival assays. a Western blot analysis of RAD52 depletion. b Representation images of CFUs from each condition after 12 days. c Quantitative analysis of colony formation. (d–f) MDA-MB-436 BRCA1+ cells were transiently transfected with control or RAD52 siRNA, with or without BRCA1 siRNA, for 48 h. Cells were plated for colony formation survival assays. d Western blot analysis of RAD52 and BRCA1 depletion. e Representation images of CFUs from each condition after 12 days. f Quantitative analysis of colony formation. Each experiment was performed ≥ 3 distinct times in triplicate. (PDF 456 kb) [file 13058_2017_912_MOESM1_ESM.pdf]

# S Fig. 2

## A SUM149PT BRCA1<sup>-/-</sup>

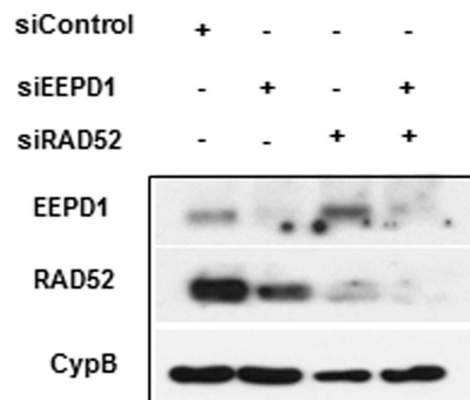

## B SUM149PT BRCA1<sup>-/-</sup>

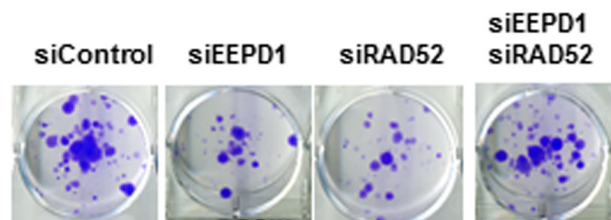

## C SUM149PT BRCA1<sup>-/-</sup>

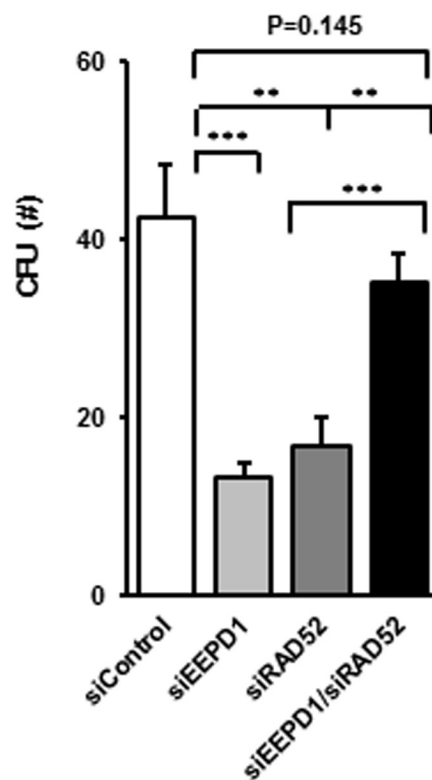

Supplement: Supplementary file 2 — EEPD1 depletion in SUM149PT BRCA1 mutant breast cancer cells rescues synthetic lethality from RAD52 depletion. a–c SUM149PT BRCA1-/- cells were transiently transfected with control or RAD52 siRNA for 48 h and cells were plated for colony formation survival assays. a Western blot analysis of RAD52 and EEPD1 depletion. b Representation images of CFUs from each condition after 12 d. c Quantitative analysis of colony formation. Each experiment was performed ≥ 3 distinct times in triplicate. (PDF 263 kb) [file 13058_2017_912_MOESM2_ESM.pdf]

# S Fig. 3

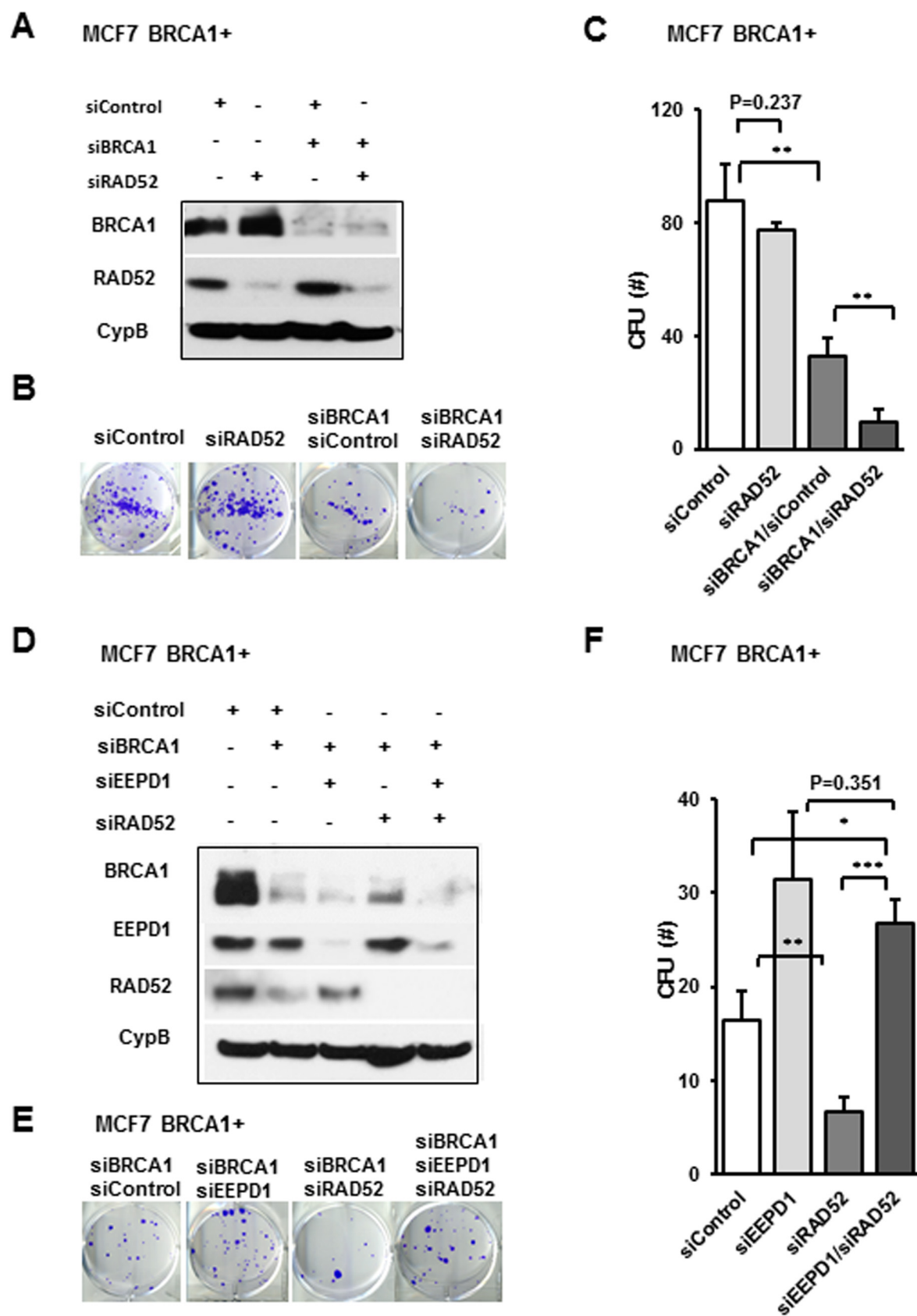

Supplement: Supplementary file 3 — EEPD1 depletion in BRCA1-depleted MCF7 breast cancer cells rescues synthetic lethality from RAD52 depletion. a–c MCF7 BRCA1-proficient cells were transiently transfected with control or RAD52 siRNA, with or without BRCA1 siRNA, for 48 h. Cells were plated for colony formation survival assays. a Western blot analysis. b Representation images of CFUs from each condition after 14 days. c Quantitative analysis of colony formation. d–f MCF7 BRCA1-proficient cells were transiently transfected with control, EEPD1 and/or RAD52 siRNA, with BRCA1 siRNA, for 48 h. Cells were plated for colony formation survival assays. d Western blot analysis. e Representation images of CFUs from each condition after 14 days. f Quantitative analysis of colony formation. Each experiment was performed ≥ 3 distinct times in triplicate. (PDF 459 kb) [file 13058_2017_912_MOESM3_ESM.pdf]

# S Fig. 4

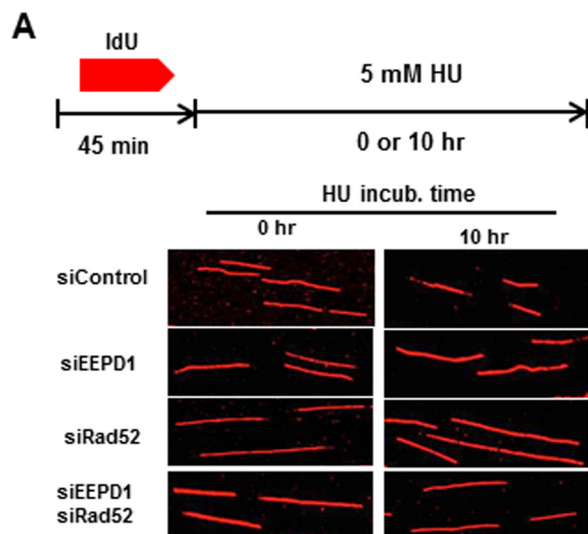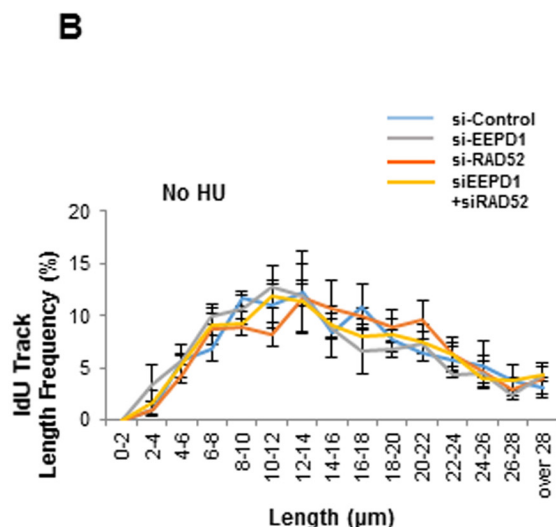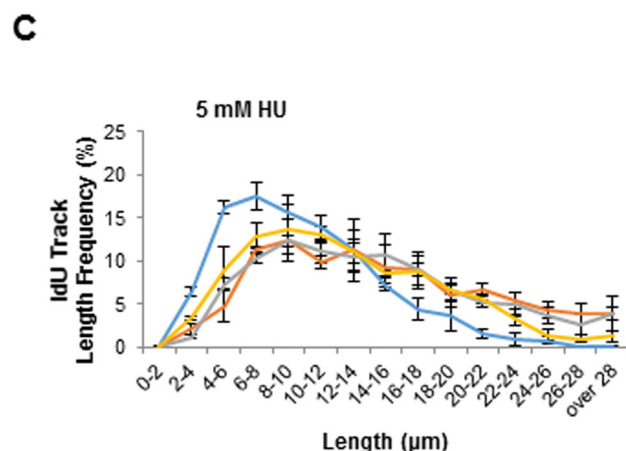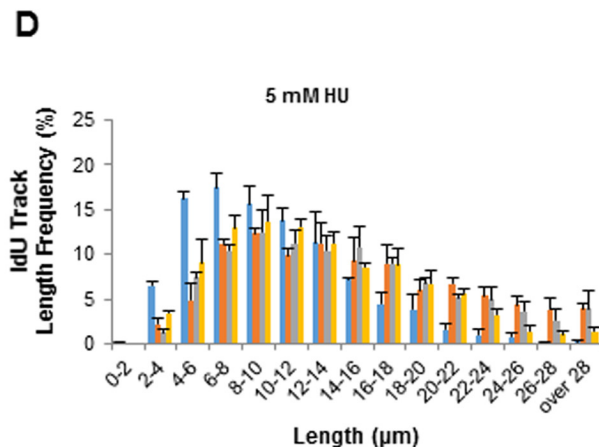

Supplement: Supplementary file 4 — Single-label DNA fiber analysis of stressed replication fork degradation. MDA-MB-436 BRCA1-/- cells were transiently transfected with control, EEPD1 and/or RAD52 siRNA for 48 h and labeled with IdU for 45 min before proceeding to either 0 h or 10 h incubation with 5 mM HU. DNA degradation at stalled nascent replication forks was measured by fiber analysis. a Schematic diagram depicts steps for the DNA fiber assay and representative images of DNA fibers from each condition. IdU stained red (stalled forks). Quantitative analysis of IdU track length frequency at unstressed (no HU) (b), or HU-treated DNA fibers (c) from each condition. d Bar chart from the HU-treated IdU track length frequencies analysis. c and d are the same data. Co-depletion of both RAD52 and EEPD1 restores stressed replication fork degradation back to control levels. Three distinct experiments per condition (>100 fibers measured per condition for each experiment). (PDF 419 kb) [file 13058_2017_912_MOESM4_ESM.pdf]
