# Supplementary material for: The endonuclease EEPD1 mediates synthetic lethality in RAD52-depleted BRCA1 mutant breast cancer cells
Source: Breast Cancer Res. 2017 Nov 16;19:122. doi: 10.1186/s13058-017-0912-8 (PMC5693420; doi:10.1186/s13058-017-0912-8)
Supplement: Supplementary file 5 — cNHEJ DNA repair pathway is nonessential for MDA-MB-436 BRCA1 mutant breast cancer cells to survive. a–c MDA-MB-436 BRCA1-/- cells were transfected with control or XRCC4 siRNA for 48 h and cells were plated for colony formation survival assays. a Western blot analysis of XRCC4 depletion. b Representation images of CFUs from each condition after 14 days. c Quantitative analysis of colony formation. d–f MDA-MB-436 BRCA1-/- cells were transfected with control or LIG4 siRNA for 48 h and cells were plated for colony formation survival assays. d Western blot analysis of XRCC4 depletion. e Representation images of CFUs from each condition after 14 days. f Quantitative analysis of colony formation. g–i MDA-MB-436 BRCA1-/- cells were transfected with control or POLQ siRNA for 48 h and cells were plated for colony formation survival assays. g Western blot analysis of POLQ depletion. h Representation images of CFUs from each condition after 14 days. i Quantitative analysis of colony formation. Each experiment was performed ≥ 3 distinct times in triplicate. (PDF 580 kb) [file 13058_2017_912_MOESM5_ESM.pdf]

S Fig. 5

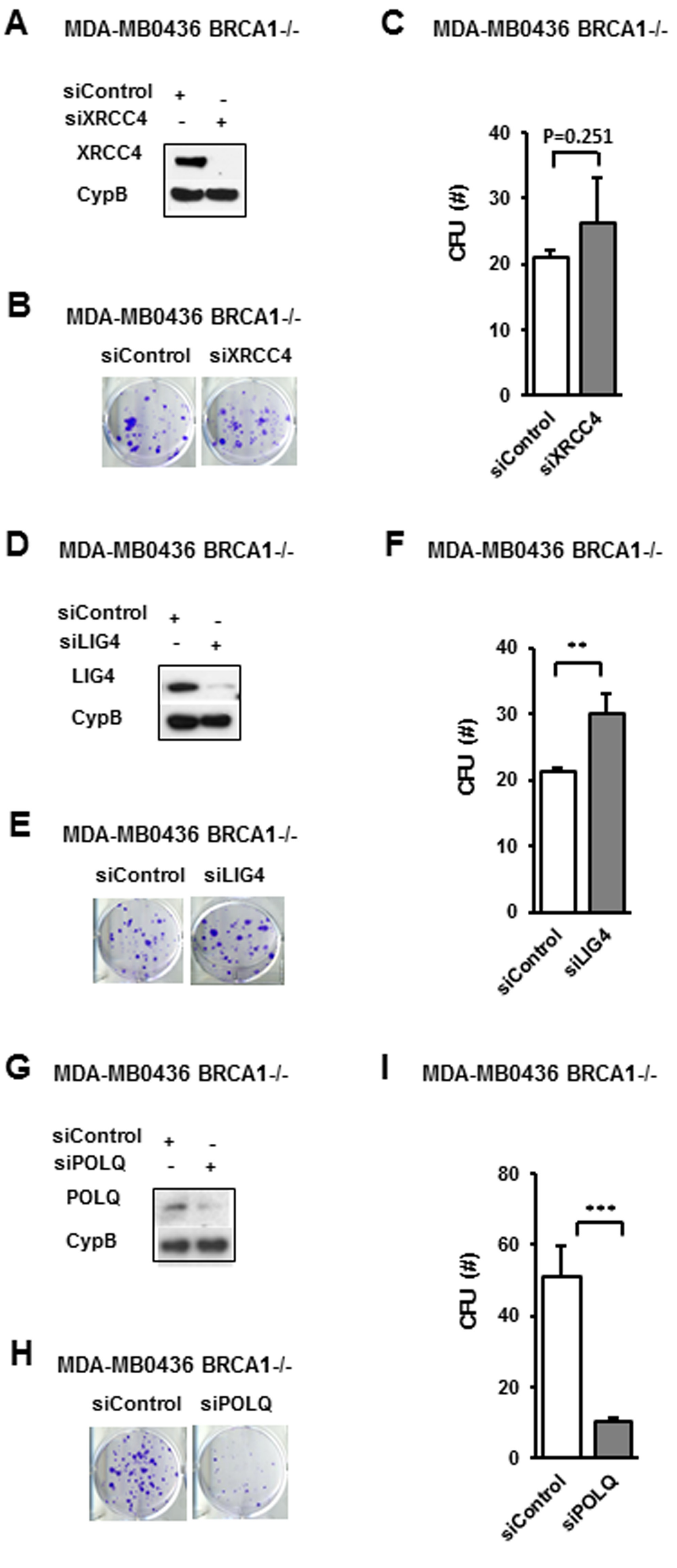
A
MDA-MB0436 BRCA1-/-
siControl + -
siXRCC4 - +
XRCC4
CypB
B
MDA-MB0436 BRCA1-/-
siControl
siXRCC4
C
MDA-MB0436 BRCA1-/-
P=0.251
CFU (#)
0
10
20
30
40
siControl
siXRCC4
D
MDA-MB0436 BRCA1-/-
siControl + -
siLIG4 - +
LIG4
CypB
E
MDA-MB0436 BRCA1-/-
siControl
siLIG4
F
MDA-MB0436 BRCA1-/-
**
CFU (#)
0
10
20
30
40
siControl
siLIG4
G
MDA-MB0436 BRCA1-/-
siControl + -
siPOLQ - +
POLQ
CypB
H
MDA-MB0436 BRCA1-/-
siControl
siPOLQ
I
MDA-MB0436 BRCA1-/-
***
CFU (#)
0
20
40
60
80
siControl
siPOLQ
